# Maternal and Infant Outcomes for Women Experiencing Homelessness Before and During Pregnancy: A Retrospective Cohort Study

**DOI:** 10.1111/1471-0528.70050

**Published:** 2025-10-10

**Authors:** Dorothea Geddes‐Barton, Raph Goldacre, Serena Luchenski, Chelsea Daniels, Rhiannon D′Arcy, Marian Knight, Nicola Vousden

**Affiliations:** ^1^ National Perinatal Epidemiology Unit, Nuffield Department of Population Health University of Oxford Oxford UK; ^2^ Applied Health Research Unit, Nuffield Department of Population Health University of Oxford Oxford UK; ^3^ Collaborative Centre for Inclusion Health, Institute of Epidemiology and Healthcare University College London London UK; ^4^ Department of Women and Children's Health School of Life Course Sciences, Faculty of Life Sciences and Medicine, King's College London London UK

**Keywords:** homelessness, inclusion health, maternal morbidity, routine data

## Abstract

**Objective:**

To explore whether women experiencing homelessness during pregnancy have higher risks of adverse pregnancy outcomes compared to housed women.

**Design:**

Population‐based retrospective cohort study using national electronic hospital records.

**Setting:**

Maternity services across English NHS hospitals.

**Population:**

Women giving birth at gestational age ≥ 24 weeks from January 1, 2013 to March 31, 2023.

**Methods:**

Data were obtained from the English National Hospital Episode Statistics Admitted Patient Care database. Poisson regression models compared outcomes for women identified as homeless to housed women, adjusting for age, parity, ethnicity, year and pre‐existing medical conditions.

**Main Outcome Measures:**

Severe maternal morbidity (SMM), preterm birth (< 37 and < 34 weeks), and low birth weight (< 2500 g).

**Results:**

Among 3 349 601 women giving birth, 3301 (0.1%) experienced homelessness. Rates and adjusted risk ratios (aRR) comparing homeless to housed women were: SMM 2.5% versus 1.6% (aRR 1.28, 95% CI 1.02–1.59); preterm birth 11.8% versus 5.9% (aRR 1.88, 95% CI 1.69–2.08); and small for gestational age 9.0% versus 4.8% (aRR 1.56, 95% CI 1.38–1.76). Stratified by ethnicity, White homeless women had the highest risk for preterm birth and small for gestational age, while Asian homeless women showed the greatest risk for SMM, compared to White housed women.

**Conclusions:**

Homelessness recorded during pregnancy or at birth is associated with poorer maternal and infant outcomes. Interventions focusing on housing stability are key. Future research should explore housing dynamics beyond homelessness, including frequent moves and overcrowding, requiring detailed perinatal housing data.

## 1 Introduction

Homelessness is not solely characterised by a lack of a roof or literal homelessness; it also encompasses insecure, unstable, and inadequate housing [1]. Safe, secure, healthy housing is a crucial determinant of health throughout life [2]. People experiencing homelessness experience high rates of psychiatric disorders, substance abuse, infectious diseases, and premature mortality [3, 4].

No routine data sources are capturing the prevalence of homelessness in pregnancy in the UK. It is estimated from state surveillance systems in the USA that 4% of women were homeless within 12 months before pregnancy [5]. A recent systematic review identified nine observational studies, all from the USA, demonstrating that homelessness in pregnancy was associated with preterm birth, low birth weight, neonatal admission to intensive care, and delivery complications [6].

People experiencing homelessness, including those who are pregnant, are more likely to experience intersecting challenges such as poverty, trauma, and social exclusion [7, 8]. Therefore, the associations of homelessness on pregnancy outcomes may be intertwined with challenges of economic deprivation, limited access to quality healthcare, lack of trust in healthcare, and stigma from practitioners [9]. Women experiencing homelessness are also less likely to receive recommended antenatal and postnatal care [5, 6]. Ethnicity may compound these risks [9]. Women from minoritised ethnicities are more likely to experience homelessness [10] and experience adverse maternal and neonatal outcomes [11]. However, the intersection of homelessness and ethnicity on pregnancy and birth outcomes is currently poorly understood.

Pregnancy offers a pivotal opportunity to engage women experiencing homelessness and develop trusting relationships to impact not just maternal experiences but also the development and lifespan trajectories of their children [12]. Homelessness is also a modifiable risk factor. Therefore, by understanding the impact of homelessness on pregnancy outcomes, the women at greatest risk of adverse outcomes could be identified to provide targeted interventions to improve outcomes. However, robust evidence from the UK is absent to inform practice and policy.

The primary aim of this study was to determine whether women experiencing homelessness in the UK are at greater risk of SMM, preterm gestation, and small for gestational age at birth. A secondary aim was to explore how any increased risk interacts with ethnicity. Patient and public involvement was undertaken to understand the acceptability of using routine health data to describe social factors such as housing and the impact of housing on pregnancy experiences.

## 2 Methods

### 2.1 Study Design

A retrospective nationwide population‐based cohort study was conducted using the English National Hospital Episode Statistics Admitted Patient Care (HES APC) database. Based on previous work, a phenotype (set of code lists) [13] was used to identify women experiencing homelessness within HES APC at the time of birth. Poisson regression adjusted for relevant confounding factors was applied to calculate the risk of severe maternal morbidity, preterm birth, small for gestational age, and low birth weight for women experiencing homelessness compared to housed women.

### 2.2 Data Source

The HES APC is a national administrative hospital database that includes records of all hospital admissions in the NHS and covers around 97% of all births in England. It contains demographics and clinical information (diagnoses and procedures). The HES APC also contains pregnancy and birth‐specific information in an additional maternity section. This study extracted data on all childbirth episodes in England between 1 January 2013 and 31 March 2023 and linked hospital admissions for any cause from 1 January 2003. Further details of the HES APC database have been described elsewhere [14]. Diagnostic information is coded using the International Classification of Disease 10th edition (ICD‐10), and operative procedures are coded using the UK Office for Population Censuses and Surveys classification, fourth revision (OPCS‐4) [13]. There is no consensus‐based agreed minimum set of outcomes that should be measured and reported for this population, and therefore, core outcome sets were not used.

### 2.3 Ethics Committee Approval, Data Availability and Reporting

Under the assessment of the NHS Health Research Authority, using the HES APC data to conduct epidemiological and health service research at the University of Oxford does not need research ethics committee approval as it is anonymised data. This study is reported according to recommendations in the RECORD Guidelines [14].

### 2.4 Population, Exposure and Outcome (See Supplementary Methods Section for More Details)

This study included all women aged 10–55 who gave birth (including live and stillbirths) between 1 January 2013 and 31 March 2023 in a hospital, with a gestational age at childbirth of ≥ 24 weeks. If a woman had more than one birth in the time period, one birth only was randomly selected to exclude the effect of clustering within individuals. Women experiencing homelessness in the dataset were identified using a phenotype for homelessness created in the HES APC database [15]. This phenotype is based on identifiers developed through consultation with individuals with lived experience of homelessness and clinical collaborators. The identifiers include women whose address is recorded as ‘no fixed abode’ (NFA) at the time of birth admission, and/or those registered at GP practices that exclusively serve homeless populations and/or those with a diagnosis that includes the ICD‐10 code for homelessness (Z59.0). The outcomes included SMM, which is defined as a serious health event for the mother around the time of birth and was identified in this study using a modified English Maternal Morbidity Outcome Indicator (EMMOI) that includes 21 diagnoses and 16 procedures [16], low birth weight < 2500 g, preterm birth, and small for gestational age (< 10th percentile) defined using Intergrowth‐21st standards [17]. Further detail on the included codes is in Table S1. There was 7.3% missing information on fetal sex, and these women were excluded from the analysis of the small for gestational age outcome.

A Directed Acyclic Graph (DAG) (Figure S1) was used to conceptually represent which variables were confounders or mediators based on both existing literature and clinical knowledge a priori. Age was modelled as a categorical variable in 5‐year age groups as there was statistically significant evidence of departure from linearity (*p* < 0.001). Ethnicity was collapsed into five subgroups: White, Black, Asian, and Other, including Mixed and Chinese. Parity was categorised into nulliparous (yes/no). Information on the history of pre‐existing medical conditions (yes/no) and psychosocial adversity (yes/no) was obtained based on ICD‐10 codes. The year is identified by the baby's birth year.

### 2.5 Statistical Analysis

Statistical analysis was performed using StataCorp. 2023. *Stata Statistical Software: Release 18*. Statistical significance was assumed to be a *p*‐value of less than 0.05. The incidence of SMM, preterm birth, small for gestational age, and low birth weight for women experiencing homelessness and housed women was calculated using the number of maternities [18] (women with either live or stillbirths) in each group as the denominator. The characteristics of the women in the study population are presented as numbers and percentages in each group stratified by housing status. Poisson regression was used to estimate SMM, preterm birth, small for gestational age, and low birth weight risk ratios and their 95% confidence intervals for women experiencing homelessness compared to housed women. Three models were built using multivariable Poisson regression to estimate the risk ratios of SMM, preterm birth, small for gestational age, and low birth weight and their 95% confidence intervals for women experiencing homelessness compared to housed women using a complete case analysis (excluding 4.6% of women with missing data on ethnicity). Models were adjusted for (1) age, ethnicity, parity, year of birth; (2) model 1 + pre‐existing medical conditions (Table S2); (3) model 2 + psychosocial adversity (which included codes for mental health problems, substance misuse, smoking, and domestic violence [19]). The characteristics of the women in the study population were stratified by four ethnic groups.

### 2.6 Secondary Analyses

All secondary analyses used multivariable Poisson regression to estimate risk ratios (and 95% CIs) for SMM, preterm birth, small for gestational age, and low birth weight, adjusting for age, parity, year, ethnicity, and pre‐existing medical conditions. The first analysis compared homeless and housed women across four ethnic groups to housed White women. The second used housed women in the least deprived areas as the reference group to better isolate the effect of homelessness beyond deprivation (excluding 2.4% of women with missing data on the Index of Multiple Deprivation [IMD]). A third analysis identified homelessness based on any hospital admission in the 2 and 5 years prior to, and including, the birth admission. An analysis further adjusted for region, comparing homeless and housed women across ethnic groups to housed White women, to account for potential regional differences in coding practices affecting minoritised ethnicities.

### 2.7 Sensitivity Analysis

As a sensitivity analysis, we conducted inverse probability weighted Poisson regression models with doubly robust adjustment, incorporating the same covariates used in the propensity score model (age, ethnicity, parity, year, and pre‐existing medical conditions). The propensity scores for homelessness were estimated using a logistic regression model. Two‐way interactions among predictors were considered; however, model comparison using Akaike information criterion (AIC), Bayesian information criterion (BIC), the Hosmer–Lemeshow goodness‐of‐fit test, and the area under the receiver operating characteristic curve (AUC) indicated that the simpler model without interactions provided the best fit. Inverse probability of treatment weights were derived from this model, and extreme weights were trimmed at the 1st and 99th percentiles to reduce the influence of outliers. Covariate balance after weighting was evaluated using standardised mean differences (SMDs), with all SMDs < 0.1 indicating adequate balance. Propensity score distributions were inspected to confirm sufficient overlap between exposed and unexposed groups.

To address potential bias from missing birthweight and gestational age data, we imputed the values for preterm birth, small for gestational age, and birthweight < 2500 g, using fully conditional specification multiple imputation by chained equations to generate 20 datasets and pool estimates using Rubin's rules [20]. We performed a second multiple imputation using the above method, imputing the values for missing fetal sex in the small for gestational age analysis. For the SMM outcome, we repeated the analysis including all women with missing gestational age and birthweight (14%).

### 2.8 Patient and Public Involvement

Before commencing this study, we held a 2‐h discussion with six women with recent lived experience of multiple social disadvantages [21] in pregnancy, including living in temporary and homeless accommodation. We explored the acceptability of using routine health data to describe social factors such as housing and deprivation and how their social circumstances, including housing and homelessness, impacted pregnancy experiences and may influence pregnancy outcomes (Box 1).

## 3 Results

### 3.1 Characteristics of the Study Population

There were 3,349,601 women who gave birth in NHS hospitals in England in this 10‐year period (Figure S2). 3303 (0.1%) women were identified as homeless using the phenotype. Table S3 shows the breakdown of the homelessness identifiers. Characteristics of the women stratified by homelessness status are shown in Table 1. A greater proportion of women experiencing homelessness resided in the most deprived areas of England (48.8% vs. 25.8%), were aged under 25 (33.3% vs. 17.1%), were of Black ethnicity (21.9% vs. 5.0%), and experienced psychosocial adversity (38.8% vs. 25.6%) compared to housed women.

**TABLE 1:** Characteristics of women by homelessness status.

	Homeless code during birth admission		
	Housed women	Women experiencing homelessness	Total
Total	3 346 298 (99.9%)	3303 (0.1%)	3 349 601 (100.0%)
IMD^a^			
Most deprived 20%	842 331 (25.2%)	1424 (43.1%)	843 755 (25.2%)
More deprived 20%–40%	745 173 (22.3%)	993 (30.1%)	746 166 (22.3%)
Less deprived 40%–60%	627 667 (18.8%)	336 (10.2%)	628 003 (18.7%)
Less deprived 60%–80%	553 863 (16.6%)	128 (3.9%)	553 991 (16.5%)
Least deprived 80%–100%	497 289 (14.9%)	60 (1.8%)	497 349 (14.8%)
Missing	79 975 (2.4%)	362 (11.0%)	80 337 (2.4%)
Age group			
10–20	103 623 (3.1%)	282 (8.5%)	103 905 (3.1%)
20–25	468 097 (14.0%)	818 (24.8%)	468 915 (14.0%)
25–30	913 995 (27.3%)	886 (26.8%)	914 881 (27.3%)
30–35	1 085 045 (32.4%)	790 (23.9%)	1 085 835 (32.4%)
35–40	622 388 (18.6%)	415 (12.6%)	622 803 (18.6%)
> 40	153 150 (4.6%)	112 (3.4%)	153 262 (4.6%)
Ethnicity			
White	2 451 450 (76.8%)	1537 (49.7%)	2 452 987 (76.8%)
Black	160 472 (5.0%)	678 (21.9%)	161 150 (5.0%)
Asian	362 996 (11.4%)	382 (12.4%)	363 378 (11.4%)
Other	217 205 (6.8%)	495 (16.0%)	217 700 (6.8%)
Missing	154 175 (4.6%)	211 (6.4%)	155 501 (4.6%)
Parity			
Multiparous	2 034 385 (60.8%)	1914 (57.9%)	2 036 299 (60.8%)
Primiparous	1 311 913 (39.2%)	1389 (42.1%)	1 313 302 (39.2%)
Year			
2013	387 452 (11.6%)	295 (8.9%)	387 747 (11.6%)
2014	371 978 (11.1%)	311 (9.4%)	372 289 (11.1%)
2015	355 459 (10.6%)	336 (10.2%)	355 795 (10.6%)
2016	347 233 (10.4%)	341 (10.3%)	347 574 (10.4%)
2017	337 349 (10.1%)	352 (10.7%)	337 701 (10.1%)
2018	317 782 (9.5%)	397 (12.0%)	318 179 (9.5%)
2019	299 815 (9.0%)	348 (10.5%)	300 163 (9.0%)
2020	288 829 (8.6%)	282 (8.5%)	289 111 (8.6%)
2021	296 933 (8.9%)	241 (7.3%)	297 174 (8.9%)
2022	280 344 (8.4%)	324 (9.8%)	280 668 (8.4%)
2023	63 124 (1.9%)	76 (2.3%)	63 200 (1.9%)
Pre‐existing medical conditions			
No	2 653 743 (79.3%)	2625 (79.5%)	2 656 368 (79.3%)
Yes	692 555 (20.7%)	678 (20.5%)	693 233 (20.7%)
Psychosocial adversity^b^			
No	2 488 460 (74.4%)	2022 (61.2%)	2 490 482 (74.4%)
Yes	857 838 (25.6%)	1281 (38.8%)	859 119 (25.6%)

^a^ IMD available for women with an ICD‐10 homelessness code or registered with a homeless GP, but missing for ‘NFA’ women.

^b^ This was a composite based on Harron et al. [19] See Table S1 for included codes.

For the total population, the overall risk of SMM, preterm birth < 37 and < 34 weeks, small for gestational age, and low birth weight at the time of childbirth was 1.6%, 5.9%, 1.6%, 5.2% and 5.8%, respectively (Table 2). The risk ratios and their 95% confidence intervals for the sequential models are shown in Table 2. Compared with housed women, women experiencing homelessness at the time of childbirth had a greater risk of SMM (1.6% vs. 2.5%, unadjusted RR 1.53 [95% CI 1.24–1.90]). The incidence of individual SMM conditions by housing status is shown in Table S4. After adjustment for age, year of birth, ethnicity, and parity, the RR attenuated slightly (Model 1 aRR 1.28, 1.02–1.60); further adjusting for pre‐existing medical conditions (Model 2) and psychosocial adversity (Model 3) made no substantive difference (Table 2).

**TABLE 2:** The association between homelessness coded during the birth episode and severe maternal morbidity (SMM), preterm birth, low birth weight, and small for gestational age (< 10th percentile) compared to all housed women in England, 2013–2023. Risk ratios (RR) and their 95% confidence intervals (CI).

	Housed women *N* = 3 346 298 (3099225^a^)	Women experiencing homelessness *N* = 3303 (3170^a^)	Univariable	Model 1 (adjusted for age, ethnicity, year, parity)	Model 2 (Model 1 + pre‐existing medical conditions)	Model 3 (Model 2 + psychosocial adversity)
	*N* (%)	*N* (%)	Risk ratio [95% CI]	Risk ratio [95% CI]	Risk ratio [95% CI]	Risk ratio [95% CI]
SMM	54 902 (1.6%)	83 (2.5%)	1.53 [1.24 1.90]	1.28 [1.02 1.60]	1.28 [1.02 1.59]	1.28 [1.02 1.60]
Gestational age at birth						
< 37 weeks	198 014 (5.9%)	390 (11.8%)	2.00 [1.81 2.20]	1.89 [1.71 2.10]	1.88 [1.69 2.08]	1.77 [1.60 1.97]
< 34 weeks	51 857 (1.5%)	123 (3.7%)	2.40 [2.01 2.87]	2.16 [1.80 2.58]	2.14 [1.78 2.56]	2.01 [1.68 2.41]
Small for gestational age	148 392 (4.8%)	284 (9.0%)	1.87 [1.67 2.10]	1.56 [1.38 1.76]	1.56 [1.38 1.76]	1.46 [1.29 1.65]
Low birth weight < 2500 g	192 782 (5.8%)	430 (13.1%)	2.27 [2.06 2.49]	1.99 [1.81 2.20]	1.98 [1.80 2.19]	1.83 [1.66 2.02]

^a^ In the small for gestational age analysis excluding 7.3% with missing fetal sex.

The proportion of women experiencing homelessness with a preterm birth < 37 weeks was 11.8% compared to 5.9% for housed women (Model 2 aRR 1.88, 1.69–2.08) and 3.7% compared to 1.5% for preterm birth < 34 weeks (Model 2 aRR 2.14, 1.78–2.56). Similarly, the proportion of small for gestational age was 9.0% for women experiencing homelessness compared to 4.8% in housed women (Model 2 aRR 1.56, 1.38–1.76). After an additional adjustment for psychosocial adversity, there was a modest reduction in the risk of preterm birth and small for gestational age in women experiencing homelessness compared to housed women, which remained significantly increased (aRR 2.01 [95% CI 1.68–2.41] and 1.46 [95% CI 1.29–1.65]) respectively, Model 3.

### 3.2 Secondary Analyses

A secondary analysis was undertaken to compare the risk of SMM, preterm birth, small for gestational age, and low birth weight between women experiencing homelessness and women living in the most deprived areas of England. The aRR remained similar, as shown in Table 3 (compared to the most deprived IMD quintile) and Table S5 (compared to the most deprived IMD decile).

**TABLE 3:** The association between homelessness coded during the birth episode and severe maternal morbidity (SMM), preterm birth, low birth weight, and small for gestational age (< 10th percentile) compared to housed women living in the most deprived quintile of England (IMD 5). Risk ratios (RR) and their 95% confidence intervals (CI).

	Housed women IMD 5^a^, *N* (%)	Women experiencing homelessness, *N* (%)	Univariable	Model 1 (adjusted for age, ethnicity, year, parity)	Model 2 (Model 1 + pre‐existing medical conditions)	Model 3 (Model 2 + psychosocial adversity)
Total	842 331 (99.8%) 782 706 (99.6%)^b^	1786 (0.2%) 3170 (0.4%)^b^				
SMM	14 701 (1.7%)	83 (2.5%)	1.44 [1.16 1.79]	1.24 [0.99 1.55]	1.23 [0.98 1.54]	1.23 [0.99 1.54]
Gestational age at birth						
< 37 weeks	58 991 (7.0%)	390 (11.8%)	1.69 [1.53 1.86]	1.71 [1.54 1.90]	1.70 [1.53 1.88]	1.62 [1.47 1.80]
< 34 weeks	16 197 (1.9%)	123 (3.7%)	1.94 [1.62 2.31]	1.90 [1.59 2.28]	1.88 [1.57 2.26]	1.80 [1.50 2.15]
Small for gestational age	47 313 6.0%	284 9.0%	1.48 [1.32 1.67]	1.41 [1.25 1.59]	1.41 [1.25 1.59]	1.34 [1.18 1.51]
Low birth weight < 2500 g	62 541 (7.4%)	430 (13.1%)	1.76 [1.60 1.93]	1.74 [1.58 1.92]	1.74 [1.57 1.91]	1.62 [1.47 1.79]

^a^ IMD 5 = Most deprived Index of Multiple Deprivation (IMD) quintile.

^b^ In the small for gestational age analysis excluding 7.3% with missing fetal sex.

**FIGURE 1:**
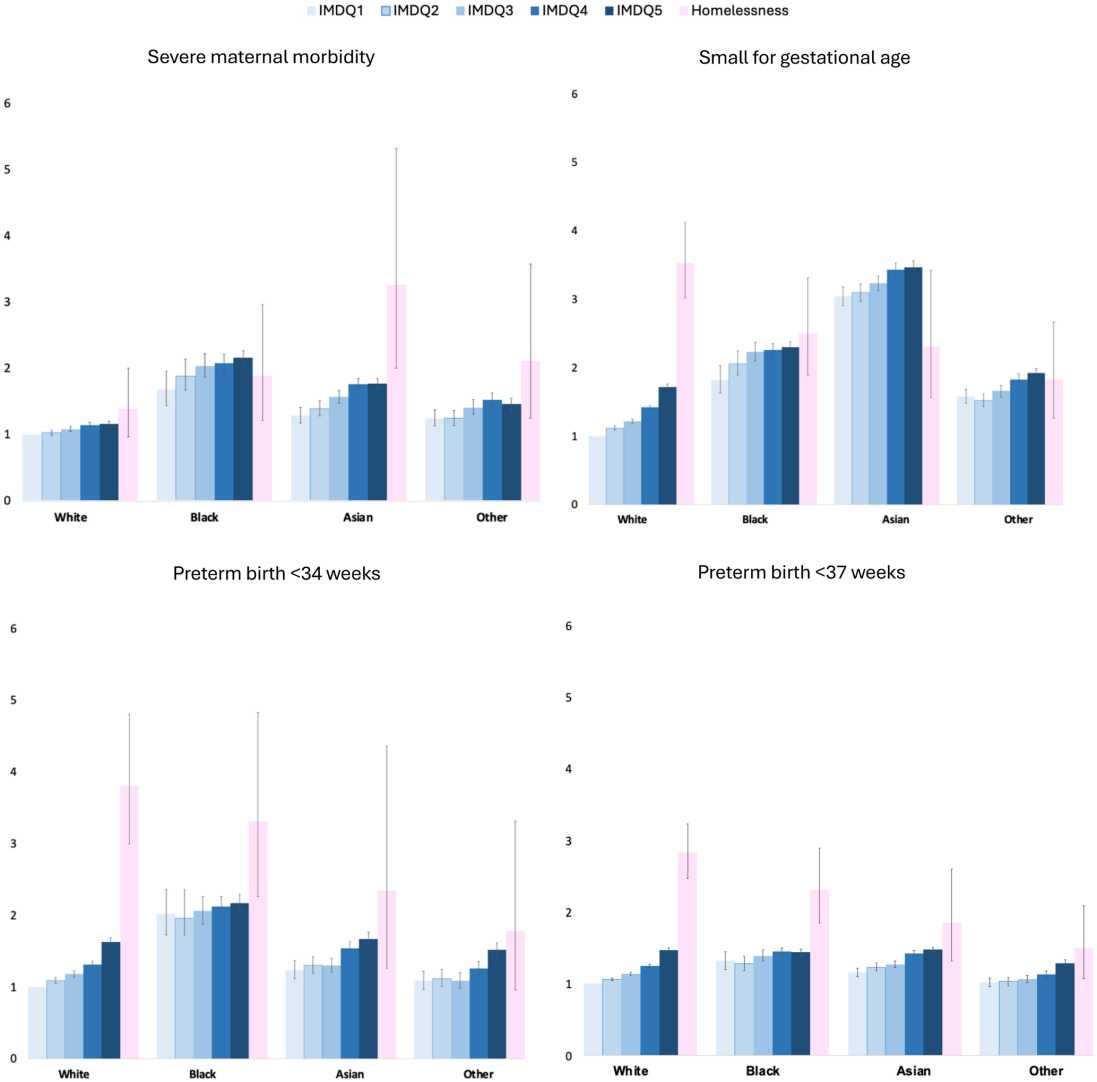
Adjusted risk ratios (RR) and their 95% confidence intervals (CI) for severe maternal morbidity, small for gestational age (< 10th centile), preterm birth < 34 weeks and preterm birth < 37 weeks, across each quintile of IMD and women experiencing homelessness. These are stratified by aggregate ethnic group compared to the least deprived white women, adjusting for age, parity, year of birth and pre‐existing medical conditions. IMDQ1 = least deprived quintile.

The characteristics of women and their housing status stratified by ethnicity are shown in Table S6. The adjusted risk of SMM was greatest in women of Asian ethnicity and homelessness, although the small numbers in these populations mean that the confidence intervals of this estimate are wide. Black women were at increased risk of SMM across all levels of deprivation, including women experiencing homelessness, compared to White women (Figure 1). There was a small reduction in the adjusted risk ratios between housed and homeless women in each ethnic group after adjusting for region, but this was not differential for housing status, the different ethnicities, or outcomes (Table S7).

The risk ratios of Model 2 (adjusting for age, parity, ethnicity, year of birth, and pre‐existing medical conditions) comparing women experiencing homelessness from any hospital admission two and five years before and including the birth admission are shown in Table S8 and show that there is a notably larger effect of homelessness on all four outcomes if homelessness is coded before the birth episode.

### 3.3 Sensitivity Analysis

There were no notable changes in the results when women with missing gestational age or birthweight were included in the cohort, including analyses using multiple imputation (Table S9). Imputing fetal sex did not change the results of the small for gestational age analysis (Table S10). The results were also consistent using inverse probability‐weighted Poisson regression (Table S11).

BOX 1 **Patient and public involvement.**

We held a group discussion with six women with recent experience of pregnancy with a background of multiple disadvantages. We explored the use of routine health data to capture social factors such as housing and homelessness and the factors around this, including financial status and relationships. The group was comfortable disclosing this information to maternity staff, and for routine data to be pooled and anonymised to improve understanding of their impact on pregnancy. The group felt there were respectful and discrete ways of asking this information and ensuring it was recorded to prevent being asked multiple times.

The women clearly described a negative impact of housing insecurity and homelessness on pregnancy. The uncertainty caused significant mental health strain, compounded by the stress of repeatedly contacting housing services. It was also difficult to constantly explain to nurses about being homeless. Prolonged hospital stays while waiting for accommodation took a toll on physical health. Moving to new accommodations late in pregnancy or after birth, often in unsuitable areas, added to the stress. Additionally, there was a lack of adequate space and equipment to properly care for the baby. This was against a background of other intertwined social factors; for example, the risk of homelessness in pregnancy being preferable to staying in an unsafe home environment at risk of domestic abuse or forced migration.

## 4 Discussion

### 4.1 Main Findings

From 3 349 601 births between 2013 and 2023, 3301 (0.1%) women experienced homelessness. More women experiencing homelessness lived in the most deprived areas of England, were younger, of Black ethnicity, and faced psychosocial adversity compared to housed women. Homelessness increased the risk of SMM, preterm birth, small for gestational age, and low birth weight compared to all housed women and housed women in deprived areas. When stratified by ethnicity, homeless Asian women had the highest risk of SMM, though all ethnic groups had a higher risk than White homeless women. However, White homeless women had a higher risk of preterm birth and small for gestational age than housed or homeless women from other ethnic groups.

### 4.2 Strength and Limitations

The main strength of this study is the use of hospital data, which covers all births in NHS hospitals in England [13], reducing selection bias and providing a large sample size. Housing status was confirmed using multiple methods to capture various forms of homelessness. A composite outcome of SMM is useful for policymakers for overall pregnancy health and quality of care during pregnancy and birth. This also avoids the limitations of evaluating rare, severe morbidities that may be more likely to be coded incorrectly.

Using our lived experience group to understand these methods' acceptability and put context onto the health impacts was vital to accelerate understanding of the results. Our lived experience group was organised through a charity known to the participants, which provided payment in line with NIHR guidance and emotional support. We have not identified any negative consequences of their involvement.

However, as data are collected not originally for research, the quality of variables around social factors such as homelessness is likely underreported [22] creating misclassification, meaning the true impact of homelessness on pregnancy outcomes may be greater or weaker. In addition, cases of street homelessness may be more likely to be identified and coded by health care professionals, so captured using this data, whereas women ‘sofa surfing’ that provide an address may be missed. Therefore, the true level of homelessness in the pregnant population may be greater. Furthermore, behaviours such as smoking or substance misuse, which are more common in homeless populations, are poorly recorded in routinely collected healthcare data, leading to unmeasured confounding. SMM is coded using ICD‐10 and OPCS‐4 codes and has not been validated using patient records, and therefore this is also a risk of false negatives of this outcome.

Women were identified as experiencing homelessness during their birth admission, but the duration of homelessness prior to this could not be determined. Homelessness recorded only at birth, as opposed to before or during any pregnancy admission, is likely the tip of the iceberg of women experiencing homelessness during pregnancy, as pregnant women are legally a ‘priority needed’ and should be offered housing [23]. However, taking homelessness at the time of birth reduces the risk of bias which may arise from measuring homelessness prior to pregnancy, as women with pre‐existing medical conditions may be more likely to have hospital admissions before pregnancy and also increase the proportion of women with SMM. Sensitivity analyses showed homelessness recorded 2–5 years before or at the birth episode had a stronger association with adverse outcomes.

### 4.3 Interpretation (In Light of Other Evidence)

Women experiencing homelessness may be at a greater risk for adverse pregnancy and birth outcomes than those who are housed due to obstacles in accessing healthcare. These barriers likely mirror those faced by the general homeless population, including denied access to primary care due to no proof of address. Other contributing factors, such as immigration status, digital exclusion, and breaks in continuity of care, may exacerbate these risks [24].

Women from minoritised ethnic groups and younger age groups are overrepresented among women experiencing homelessness. Black women experienced higher SMM risk in both women experiencing homelessness and housed populations. This aligns with literature from the USA, which has found that Black and Native American women, as well as younger women, are more likely to experience homelessness during pregnancy [25]. Addressing homelessness in pregnant women requires an intersectional framework [26], which considers how ethnicity, age, gender, and housing status interact to create compounded disadvantages. The findings of our study remained after accounting for geographical regions, such as London, where more Black women live and which may also have a higher recording of SMM conditions.

White homeless women had the highest rates of preterm birth, low birth weight, and small for gestational age, contrasting with general trends where minoritised ethnicity women face greater risks [27]. This may reflect higher levels of psychosocial adversity, such as mental health issues and trauma, and related lifestyle and health‐seeking behaviours, which may not be fully captured using routinely collected secondary care data but could be linked to adverse perinatal outcomes. These findings suggest that the pathways to homelessness, which can vary by ethnicity [28, 29], may also contribute to differential perinatal risks.

This analysis shows that most individuals are identified as homeless through ICD‐10 codes rather than “No Fixed Abode” (NFA) coding. In contrast, a study found that ‘NFA’ had the highest coded admissions among all homeless identifiers. There is limited evidence to aid in the interpretation of this finding, which could suggest gender differences: men might use NFA more frequently while women may list a temporary address. This indicates varying needs between homeless women and men. Qualitative evidence [30] suggests women's homelessness experiences are often ‘hidden’, as they are less likely to be street homeless and more likely to seek ‘safer’ spaces, such as engaging in sex work for accommodation. Moreover, women might hesitate to disclose their homelessness due to fears about child services or personal safety.

This study has highlighted the need for a more effective measure of the dynamic nature of homelessness in the data, which correctly identifies women using temporary accommodation, moving frequently, and living in poor‐quality and unsafe housing. This would help explore the true impact of homelessness on pregnancy outcomes and identify causal pathways, which are critical for designing effective interventions. Research from Canada shows that using professional, legally mandated coders increased homelessness records by 30%–40%, showing that inadequate coding likely underrepresents homelessness [31].

Finally, additional research is needed to understand how interventions aimed at the general homeless population could be adapted to the needs of pregnant women experiencing homelessness, including services such as substance misuse treatment, case management, and mental health support.

## 5 Conclusion

Pregnant women are supposed to be given priority in accessing housing under English housing law [24]. However, challenges with housing provision are widespread [32]. This paper provides evidence of the negative association between homelessness and maternal and neonatal outcomes and therefore, interventions that support women into stable and secure housing are key. Preventing intergenerational poverty, adversity, childhood maltreatment, and homelessness must begin at birth, requiring greater investment to ensure these infants are supported. Quality research is needed to investigate not only dichotomous homelessness, but also the effects of frequent moves during pregnancy, housing quality, and overcrowding. This requires detailed housing information during pregnancy and the postnatal period.

## Author Contributions

N.V. and D.G.‐B. conceptualised the project and undertook the literature review with support from C.D. N.V., D.G.‐B., S.L., and R.G. contributed to study design. D.G.‐B., R.G., and N.V. analysed the data, and all authors contributed to data interpretation and writing. All authors accept responsibility for the paper as published.

## Ethics Statement

Under the assessment of the NHS Health Research Authority, using the HES APC data to conduct epidemiological and health service research at the University of Oxford does not need research ethics committee approval as it is anonymised data.

## Conflicts of Interest

The authors declare no conflicts of interest.

## Supporting information


**Data S1:** bjo70050‐sup‐0001‐supinfo.docx.

## Data Availability

Data may be obtained from a third party and is not publicly available. The data extract was derived from the English National Hospital Episode Statistics Admitted Patient Care (HES‐APC) database with linkage to national mortality civil registrations (https://digital.nhs.uk/data‐and‐information/data‐tools‐and‐services/dataservices/linked‐hes‐ons‐mortality‐data). Linked HES‐APC and mortality data are available upon application to NHS England (formerly NHS Digital).
